# Blood‐circulating EV‐miRNAs, serum TARC, and quantitative FDG‐PET features in classical Hodgkin lymphoma

**DOI:** 10.1002/jha2.432

**Published:** 2022-04-28

**Authors:** Esther E. E. Drees, Julia Driessen, Gerben J. C. Zwezerijnen, Sandra A. W. M. Verkuijlen, Jakoba J. Eertink, Monique A. J. van Eijndhoven, Nils J. Groenewegen, Andrea Vallés‐Martí, Daphne de Jong, Ronald Boellaard, Henrica C. W. de Vet, Dirk M. Pegtel, Josée M. Zijlstra

**Affiliations:** ^1^ Amsterdam UMC Location Vrije Universiteit Amsterdam Department of Pathology Boelelaan Amsterdam The Netherlands; ^2^ Cancer Center Amsterdam Imaging and Biomarkers Amsterdam The Netherlands; ^3^ Amsterdam UMC location University of Amsterdam Department of Hematology LYMMCARE (Lymphoma and Myeloma Center) Meibergdreef Amsterdam The Netherlands; ^4^ Amsterdam UMC Location Vrije Universiteit Amsterdam Department of Radiology and Nuclear Medicine Boelelaan Amsterdam The Netherlands; ^5^ Amsterdam UMC Location Vrije Universiteit Amsterdam Department of Hematology Boelelaan Amsterdam The Netherlands; ^6^ Exbiome B.V. Amsterdam The Netherlands; ^7^ Amsterdam UMC Location Vrije Universiteit Amsterdam Department of Epidemiology and Data Science Amsterdam Public Health research institute Boelelaan Amsterdam The Netherlands

**Keywords:** extracellular vesicles, FDG‐PET, Hodgkin lymphoma, miRNAs, Radiomics, TARC

## Abstract

Blood‐based biomarkers are gaining interest for response evaluation in classical Hodgkin lymphoma (cHL). However, it is unknown how blood‐based biomarkers relate to quantitative ^18^F‐FDG‐PET features. We correlated extracellular vesicle‐associated miRNAs (EV‐miRNA), serum TARC, and complete blood count (CBC) with PET features (e.g., metabolic tumor volume [MTV], dissemination and intensity features) in 30 cHL patients at baseline. EV‐miR127‐3p, EV‐miR24‐3p, sTARC, and several CBC parameters showed weak to strong correlations with MTV and dissemination features, but not with intensity features. Two other EV‐miRNAs only showed weak correlations with PET features. Therefore, blood‐based biomarkers may be complementary to PET features, which warrants further exploration of combining these biomarkers in prognostic models.

## 1 INTRODUCTION

Classical Hodgkin lymphoma (cHL) is a lymphoid malignancy characterized by a minority of large multinucleated Hodgkin and Reed‐Sternberg (HRS) tumor cells, surrounded by a nonmalignant immune cell infiltrate [1]. Despite high survival rates in young patients, treatment of cHL relies on multidrug anthracycline‐based chemotherapy, which causes considerable burden of acute and late toxicities, such as infertility and cardiovascular toxicity [2]. ^18^F‐fluorodeoxyglucose (FDG) positron emission tomography (PET)/computed tomography (CT) imaging is performed for staging and response evaluation to guide treatment in cHL, but the positive predictive‐value for response evaluation with PET/CT is rather low [3]. Improving risk‐stratification and response assessment could be helpful to early identify chemotherapy refractory patients and switch to novel therapies [4). Molecular‐based blood‐based biomarkers can be repeated on a regular basis during treatment, which is therefore an attractive strategy to improve response assessment.

HRS cells are unique in the pathogenesis of cHL and secrete several cytokines and chemokines that actively recruit immune cells to the tumor microenvironment, which may support tumor growth [5, 6]. The CC chemokine CCL17 or thymus and activation‐regulated chemokine (TARC) is present in lymph node biopsies in approximately 90%–95% of cHL cases and is secreted by HRS cells. Serum TARC (sTARC) can be used as an early response marker in cHL and has been shown to correlate with metabolic tumor volume (MTV) [7, 8]. Noncoding microRNAs (miRNAs) in extracellular vesicles are actively secreted in the circulation by tumor cells and can potentially be used as diagnostic and prognostic cancer biomarkers [9]. In cHL patients, several miRNAs have been detected in the plasma that correlate with active disease [10]. In a previous study, we demonstrated that several miRNAs bound to small extracellular vesicles (EVs) are stably enriched in plasma of cHL patients and correlate with treatment response [11, 12].

Quantitative analysis of PET scans also provides a way to improve risk‐stratification and response assessment, complementary to the visual interpretation of the PET scan. Quantitative PET features include the assessment of MTV, which has already been shown to have prognostic value in newly diagnosed as well as relapsed/refractory (R/R) cHL patients [13, 14, 15]. Standard uptake value (SUV) features represent the intensity of glucose metabolism, and distance (dissemination) features can assess spread of disease.

The high potential of blood‐based biomarkers to improve response assessment in cHL treatment urges us to investigate how blood‐based biomarkers relate to quantitative PET features. Therefore, we aimed to assess the correlations between several experimental biomarkers (i.e. EV‐miRNAs, sTARC) and PET features in newly diagnosed and R/R cHL patients.

## 2 METHODS

**FIGURE 1:**
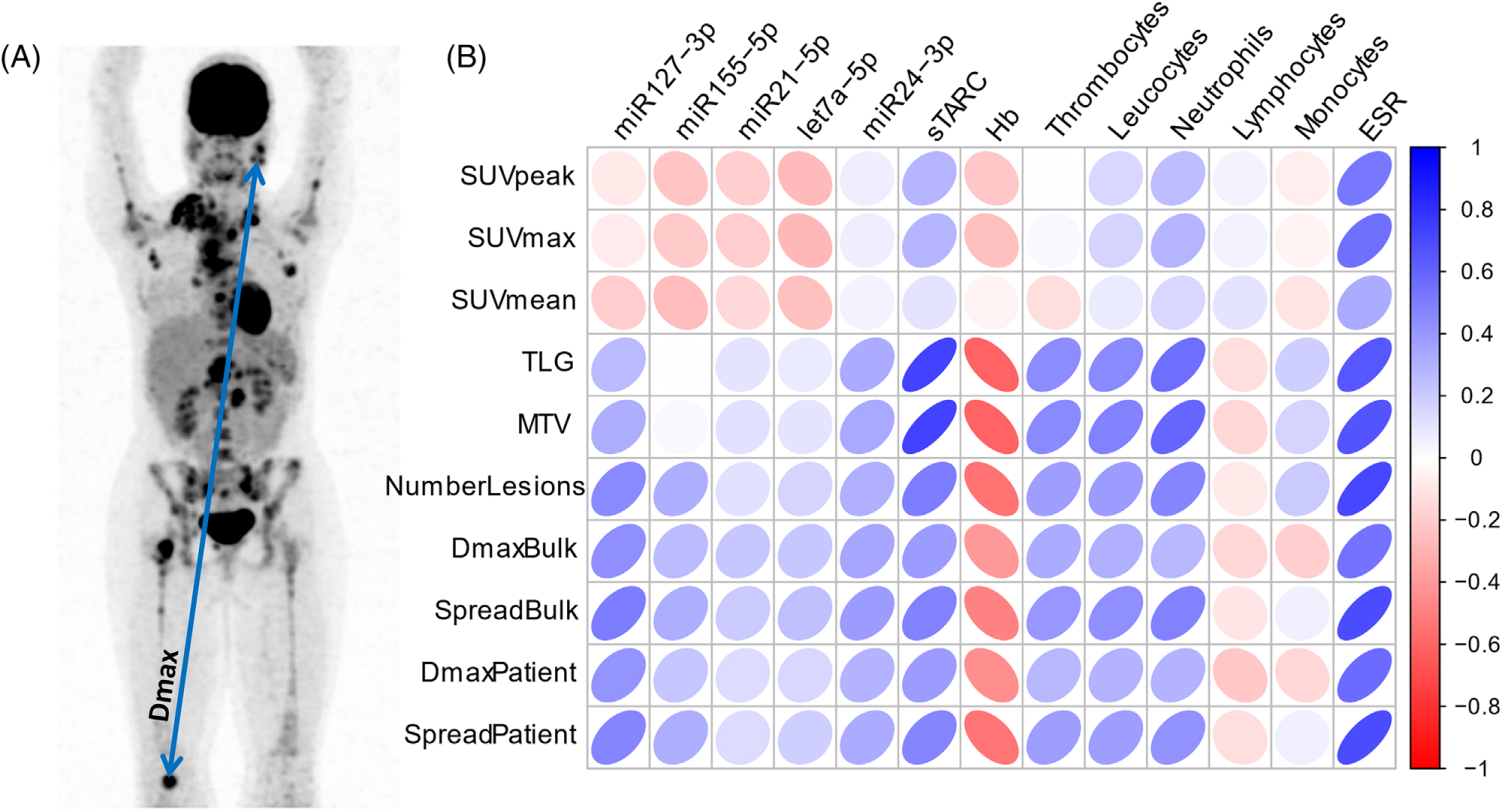
Correlation of baseline positron emission tomography (PET) features with blood‐based biomarkers. **(A)** Example of a maximum intensity projections of a PET‐scan of a newly diagnosed cHL patient. (**B)** Correlation matrix of Spearman's rank correlation coefficients between PET features and blood‐based biomarkers. ESR, erythrocyte sediment rate; Hb, hemoglobin; MTV, metabolic tumor volume; SUV, standard uptake value; TLG, total lesion glycolysis

Thirty cHL patients (*N* = 17 newly diagnosed and 13 R/R patients) who have been treated at Amsterdam UMC, were included in this comparative analysis. Patient characteristics are summarized in Table S1. PET/CT scans were performed, and blood samples were collected at baseline, that is, before start of first or salvage therapy. PET/CT scans were assessed according to the guidelines of the European Association of Nuclear Medicine [16]. MTV was calculated using semi‐automatic segmentation with a fixed threshold of SUV ≥4.0 with ACCURATE software, which has been validated in a large cohort of cHL patients [17, 18]. The following quantitative PET features were extracted from the original images: MTV, SUVmax, SUVpeak (i.e., the 1 ml with the highest SUV), SUVmean, and total lesion glycolysis (TLG; i.e. the MTV multiplied by SUVmean). Dissemination features (i.e., number of lesions, DmaxPatient, DmaxBulk, SpreadPatient, and SpreadBulk) were extracted using RaCat software [19]. The number of lesions is defined as the number of separate metabolically active lesions, DmaxPatient is the largest distance between two lesions, DmaxBulk is the largest distance between the largest lesion and one other lesion, SpreadPatient is the sum of the distances between all lesions, and SpreadBulk is the sum of the distances between the largest lesion and all other lesions (Figure 1A).

Serum and plasma samples were collected at baseline and analyzed for sTARC and plasma EV‐miRNA levels. ELISA‐based detection of sTARC was performed on serum. EV‐miRNAs were detected with qRT‐PCR as previously described [11]. Additional protocols are described in the supplemental methods. Of note, miRNA and sTARC results of a part of the current patient cohort have been published previously [11].

Wilcoxon rank sum test for nonparametric data was used to compare PET features on the baseline PET scans, between newly diagnosed and R/R patients, and to compare blood‐based biomarkers between patients with a high or low MTV and number of lesions. The median MTV (94 mL) and median number of lesions (n = 8) were chosen as cutoff for high or low MTV and number of lesions, respectively. Correlations between blood‐based parameters and PET features of the matched PET/CT scan were assessed using Spearman's rank correlation coefficients. Statistical analysis was performed using R software version 4.0.3. A *p*‐value of <0.05 was considered statistically significant.

## 3 RESULTS

Baseline PET scans of both newly diagnosed and R/R patients were included in this analysis. As such, we first analyzed differences in baseline PET features between newly diagnosed and R/R patients. MTV, TLG, and SUVpeak were significantly higher in the newly diagnosed versus R/R patients (Figure S1A). Assessing the blood‐based biomarkers, sTARC was significantly higher in the newly diagnosed patients compared to R/R patients. EV‐miRNAs were not significantly different between these groups, as previously described [11]. Further analyses were performed on the whole dataset without discriminating between newly diagnosed or R/R patients.

Correlations between PET features and complete blood‐count parameters revealed moderate to strong negative correlations between hemoglobin and several PET features including MTV, TLG, number of lesions, and Dmax and spread (Figure 1B). Thrombocyte, leucocyte, and neutrophil counts showed a weak to moderate positive correlation with MTV, TLG, number of lesions, and SpreadBulk. Erythrocyte sedimentation rate (ESR) showed a moderate to strong correlation with all intensity and dissemination features, except for SUVmean (weak correlation, *r* = 0.32). Next, we correlated the PET features with the more novel and experimental blood‐based biomarkers sTARC and EV‐miRNAs. miR127‐3p moderately correlated with the number of lesions (*r* = 0.46), SpreadPatient (*r* = 0.48), and SpreadBulk (*r* = 0.51), DmaxBulk (*r* = 0.44), and DmaxPatient (*r* = 0.42). miR24‐3p showed a weak correlation with all dissemination features. sTARC strongly correlated with MTV (*r* = 0.73) and TLG (*r* = 0.74) and weakly to moderately correlated with number of lesions (*r* = 0.51), SpreadBulk (*r* = 0.49), SpreadPatient (*r* = 0.47), DmaxBulk (*r* = 0.39), and DmaxPatient (*r* = 0.30).

**FIGURE 2:**
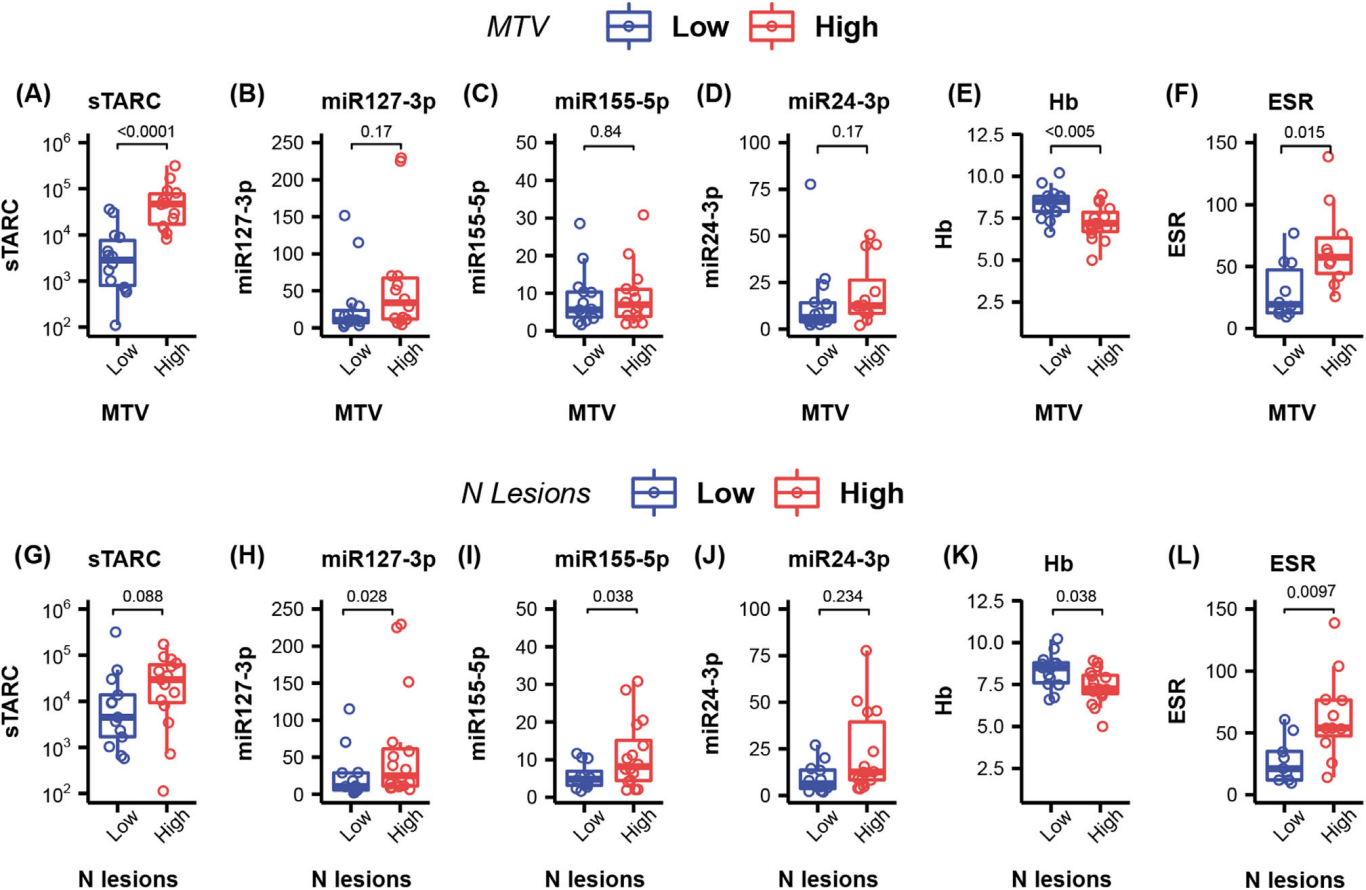
Correspondence of number of lesions and metabolic tumor volume (MTV) with levels of several blood‐based biomarkers. **(A–F)** Boxplots of several blood‐based biomarker levels, stratified for high (≥94 ml), or low (<94 ml) MTV measured by positron emission tomography (PET)/computed tomography (CT). (**G–L)** Boxplots of several blood‐based biomarker levels, stratified for a high (≥8) or low (<8) number of lesions on the PET/CT scan. Each individual dot represents a PET value of one patient, the boxplot represents the median and interquartile range, and the whiskers represent the minimum and maximum values

Median values of MTV (94 ml) and number of lesions [8] were chosen as cutoff to compare groups. sTARC levels were significantly higher for patients with a high MTV compared to patients with a low MTV, but there were no significant differences in sTARC levels for patients with a high or low number of lesions (Figure 2). None of the EV‐miRNAs showed a significant difference between patients with a low or high MTV. However, miR127‐3p and miR155‐5p levels were significantly higher in patients with a high number of lesions (*p* = 0.03 and *p* = 0.04, respectively). Hemoglobin levels were significantly lower for patients with a high MTV (*p* < 0.005). ESR was significantly higher for patients with a high MTV (*p* = 0.015).

## 4 DISCUSSION

Blood‐based biomarkers are of interest as an auxiliary tool for treatment monitoring and guidance in cHL and can possibly be used complementary to PET/CT‐based monitoring. Here, we explored the relation between several EV‐miRNAs and sTARC levels and quantitative PET features at baseline in both newly diagnosed and R/R cHL patients. We discovered several moderate to strong correlations between EV‐miR127‐3p, EV‐miR24‐3p, sTARC, and several complete blood count parameters with MTV and dissemination PET features, but there were no or only very weak correlations with intensity features.

We observed significant weak to moderate negative correlations between hemoglobin and MTV and weak to moderate positive correlations between thrombocyte, leucocyte, and neutrophil counts with MTV (Figure 1). Thus, in patients with high tumor volumes (MTV), blood abnormalities like anemia, increased thrombocytes, increased white blood cell count, and high ESR are more prevalent.

Previously, we found that the five aforementioned EV‐miRNAs are elevated in cHL when there is metabolically active disease present but are strongly reduced in complete metabolic responders [11, 12]. Here, we found that EV‐miRNAs miR21‐5p and let7a‐5p have no correlations with quantitative PET features. However miR127‐3p and miR24‐3p weakly to moderately correlated with the number of lesions and SpreadPatient, but not with MTV. Thus, even though miR21‐5p, miR155‐5p, and let7a‐5p are elevated in cHL, their levels in the blood do not correlate with the MTV. This suggests that these EV‐miRNAs are probably more immune‐response related than HRS‐cell associated, while miR127‐3p and miR24‐3p levels may be actively released in the blood stream by HRS cells [10, 20]. Likewise sTARC is HRS‐cell associated [21] and strongly correlated with the size of the tumor lesions (MTV, Figures 1 and 2) as previously reported by others [22]. So, detecting the presence of small tumor lesions in case of relapse or minimal residual disease will be more difficult based on sTARC alone.

Because the EV‐miRNAs and sTARC relate to different aspects of tumor biology, that is, supporting growth and attracting nonmalignant immune cells, combining these markers may overcome limitations of a single analyte strategy. Imaging provides insight into extension and localization of disease. Therefore correlating FDG‐PET imaging and blood‐based biomarkers is informative. The results of this study suggest that blood‐based markers, EV‐miRNA, and sTARC are differently related to image‐based biomarkers and reflect other features of the tumor biology. In conclusion, blood‐based biomarkers may be complementary to FDG‐PET in predicting treatment response. This warrants further exploration of combining these image – and blood‐based biomarkers in prognostic models.

## CONFLICT OF INTEREST

Dirk Michiel Pegtel is co‐founder and CSO of Exbiome BV and has received travel compensation from Takeda.

## AUTHOR CONTRIBUTIONS

EEED, DDJ, JMZ, and DMP contributed to the study. Study was designed with the help of MAJVE and NJG. EEED, NJG, and MAJVE performed the EV‐miRNA experiments. EEED. SAWMV, and JMZ collected the clinical data and managed the sample collection. EEED, AV, and TJM performed TARC assays. EEED and JD performed the MTV analyses, and GJCZ reviewed the analysis as second reviewer. RB provided input and supervision on the MTV analyses. JD performed the radiomics analysis. EEED and JD performed statistical analysis with the help of HCWdV. EEED, JD, GJCZ, and JMZ analyzed the data. EEED and JD wrote the manuscript with the contributions from GJCZ and JMZ. All authors reviewed the manuscript prior to submission.

## ETHICS STATMENT

Samples were collected in the BioLymph‐study (VUmc METc registration number: 2017.008; funding granted by the Dutch Cancer Society). The study was registered in the Dutch CCMO‐register (toetsingonline.nl, NL60245.029.17) and is being conducted in accordance to the Declaration of Helsinki (7th revision, October 2013) and in accordance with the Medical Research Involving Human Subjects Act (WMO). A second set of samples, prior to the BioLymph study, has been collected through biobanking and are registered at the approval committee of VUmc, Amsterdam (2018.359). Data requests can be submitted to j.zijlstra@amsterdamumc.nl.

## Supporting information

Supporting Information

Supporting Information
