# Supplementary material for: Blood‐circulating EV‐miRNAs, serum TARC, and quantitative FDG‐PET features in classical Hodgkin lymphoma
Source: EJHaem. 2022 Apr 28;3(3):908–12. doi: 10.1002/jha2.432 (PMC9422001; doi:10.1002/jha2.432)
Supplement: Supplementary file 1 — Supporting Information [file JHA2-3-908-s001.pptx]

## Slide 1
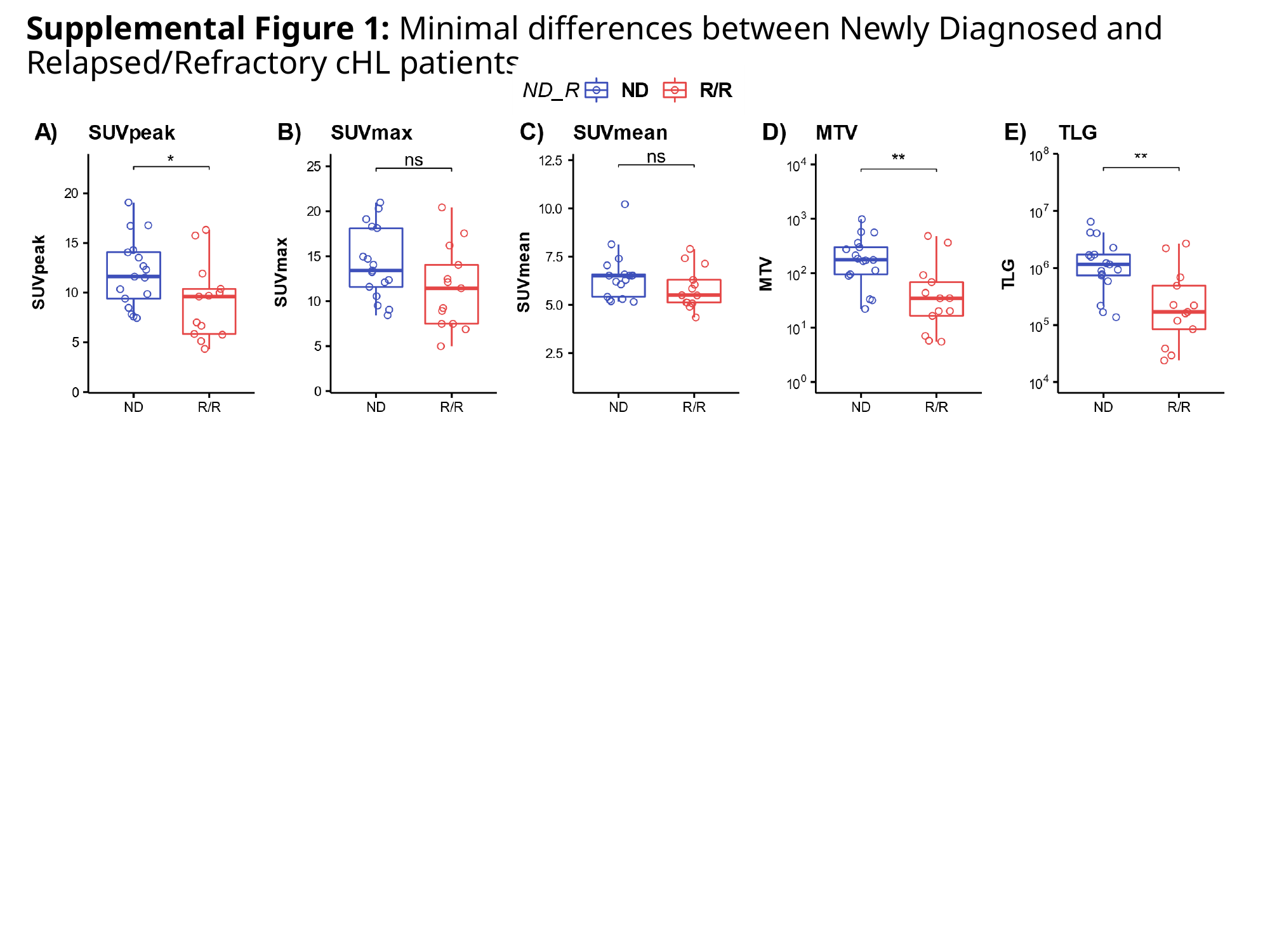

Supplemental Figure 1: Minimal differences between Newly Diagnosed and Relapsed/Refractory cHL patients

## Slide 2
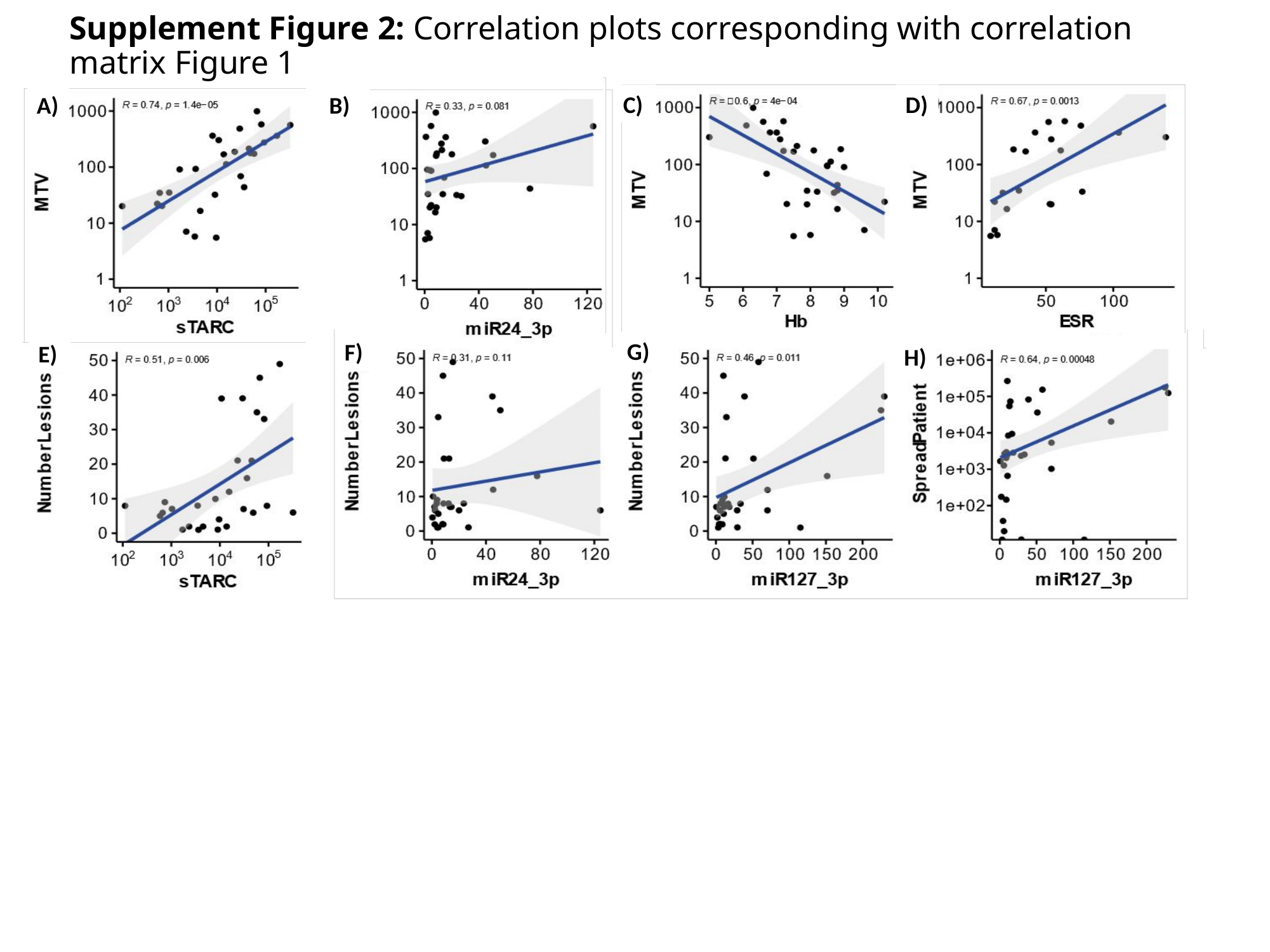

# Supplement Figure 2: Correlation plots corresponding with correlation matrix Figure 1
C)
D)
A)
B)
G)
F)
E)
H)
