# Supplementary material for: Blood‐circulating EV‐miRNAs, serum TARC, and quantitative FDG‐PET features in classical Hodgkin lymphoma
Source: EJHaem. 2022 Apr 28;3(3):908–12. doi: 10.1002/jha2.432 (PMC9422001; doi:10.1002/jha2.432)
Supplement: Supplementary file 2 — Supporting Information [file JHA2-3-908-s002.docx]

##### **Supplemental Table 1: Patient characteristics**

| Sample cohort | N = 30 |
| --- | --- |
| **Age** |  |
| Min - Max | 18 - 66 |
| Mean | 36 |
| **GHSG risk group classification system** |  |
| Limited | 6 |
| Intermediate | 6 |
| Advanced | 18 |
| **Newly diagnosed, Relapsed / Refractory** |  |
| Newly diagnosed | 17 |
| Relapsed / Refractory | 13 |
| **Treatment** |  |
| A(B)VD + IFRT | 4 |
| 2BEACOPP + 2ABVD + IFRT | 2 |
| BEACOPPesc | 11 |
| *2nd line* | |
| BV-DHAP + Beam + autologous SCT ^1^ | 7 |
| (R) DHAP + Beam + autologous SCT | 2 |
| BEACOPPesc | 1 |
| *3rd line or up* | |
| Pembrolizumab / Nivolumab | 2 |
| BV + nivolumab | 1 |

**Supplemental Table 1: Sample cohort in the analysis**

GHSG = German Hodgkin Study Group; IFRT = involved field radiotherapy; ABVD = doxorubin, bleomycine, vinblastine, dacarbazine; DHAP = dexamethasone, high-dose cytarabine, cisplatin; BEAM = carmustine, etoposide, cytarabine, melphalan; SCT = stem cell transplantation; BEACOPPesc = escalated dose of bleomycin, etoposide, doxorubicin, cyclophosphamide, vincristine, procarbazine, prednisolone; R = rituximab; BV = brentuximab Vedotin.

**Supplemental Figure legends**

**Supplemental Figure 1:** Minimal differences between Newly Diagnosed and Relapsed/Refractory cHL patients

Boxplots stratified for Newly Diagnosed (ND) versus Relapsed/Refractory (R/R). On the y-axis SUVpeak (**A),** SUVmax **(B)** MTV in ml **(C)**, TLG **(D)** and SUVmean **(E)**. *P<0.05, **P<0.01, ***P<0.001, ****<0.0001 (Mann-Whitney test). Each scan is shown as individual dot in the boxplots. line in the box plots is the median, horizontal whiskers are the range.

**Supplemental Figure 2:** Correlation plots corresponding with correlation matrix Figure 1

**A)** Correlations between MTV and sTARC, miR24-3p and miR155-5p. **B)** Correlation between number of lesions and sTARC and miR127-3p. R is Spearman rank correlation coefficient.

**Supplemental methods:**

##### **Plasma and serum collection**

Blood samples were collected in plasma collection tubes (EDTA BD Vacutainer 6 ml) and serum collection tubes (BD Vacutainer SSTII^TM^ Advance 6 ml). Within 1.5 hours of collection, isolation of poor platelet plasma and serum was performed. First, the EDTA tube was processed at 900g for 7 minutes, then supernatant was spun at 2500g for 10 minutes and in some cases an additional spin of 500g for 10 minutes was performed. Serum tubes were processed between 30 minutes and 2 hours after collection by centrifuging at 1710g for 10 minutes and then supernatant was spun at 500g for 10 minutes. Aliquots were stored at -80°C until further processing.

##### **FDG-PET CT Imaging data**

FDG-PET‐CT studies were performed using an integrated PET‐CT device (Philips Ingenuity TF, Philips Gemini TF 16). FDG-PET imaging was performed following standard clinical protocol^2^. FDG-PET scans were performed at staging, according to EANM/EARL guidelines^2^. All patients fasted for at least 6 hours prior to FDG injection and blood glucose was confirmed to be below 11 mmol/l. Metabolic tumor volume (MTV) measurements were performed using the Accurate tool^3^ and were based on the SUV≥4.0 threshold method^4^.

##### **Extracellular vesicle associated microRNA measurements**

Isolation and detection of EV-miRNAs was performed as described previously^5^ .

##### **TARC analysis**

Serum CCL17/Thymus and activation-regulated chemokine (TARC) was measured using a double antibody sandwich ELISA (Human CCL17/TARC DuoSet ELISA; R&D Systems Europe, Ltd., Abingdon, OX, UK cat#DY364, lot# P129843 and P168719) following standard protocol.

Statistics
To correct for different plasma inputs (1.0 or 1.5 ml), EV-miRNA qRT-PCR data was normalized to the mean of the complete (metabolic) response (CR) during and post treatment. Relative change is calculated using 2^^-∆∆Ct^ in where the ∆∆Ct is the difference between the Ct-value measured at that timepoint minus the Ct-value of the CR group. sTARC was measured in duplicate and the mean of the measurements is used in the analysis. Correlations between blood-based parameters and PET features of the matched PET-CT scan were assessed using Spearman rank coefficients correlation. Statistical analysis was performed using R software version 4.0.3. A P-value of <0.05 was considered statistically significant. Spearman correlation matrix was obtained using corrplot R package^6^. Analyses were performed using Graphpad Prism 9.0.1 software or R software version 4.0.3.

**References supplemental method section:**

1. Kersten MJ, Driessen J, Zijlstra JM, et al. Combining brentuximab vedotin with dexamethasone, high-dose cytarabine and cisplatin as salvage treatment in relapsed or refractory Hodgkin lymphoma: the phase II HOVON/LLPC Transplant BRaVE study. *Haematologica*. 2021;106(4):1129-1137. doi:10.3324/haematol.2019.243238

2. Boellaard R, Delgado-Bolton R, Oyen WJG, et al. FDG PET/CT: EANM procedure guidelines for tumour imaging: version 2.0. *Eur J Nucl Med Mol Imaging*. 2015;42(2):328-354. doi:10.1007/s00259-014-2961-x

3. Boellaard R. Quantitative oncology molecular analysis suite: ACCURATE. *J Nucl Med*. 2018;6:28.

4. Driessen J, Zwezerijnen GJC, Schöder H, et al. The impact of semi-automatic segmentation methods on metabolic tumor volume, intensity and dissemination radiomics in &lt;sup&gt;18&lt;/sup&gt;F-FDG PET scans of patients with classical Hodgkin lymphoma. *J Nucl Med*. January 2022:jnumed.121.263067. doi:10.2967/jnumed.121.263067

5. Drees EEE, Roemer MGM, Groenewegen NJ, et al. Extracellular vesicle miRNA predict FDG-PET status in patients with classical Hodgkin Lymphoma. *J Extracell Vesicles*. 2021;10(9). doi:10.1002/jev2.12121

6. Wei T, Simko V. R package “corrplot”: Visualization of a Correlation Matrix (Version 0.84). *R Packag*. 2017. https://github.com/taiyun/corrplot.
